# S3-Leitlinie Schilddrüsenkarzinom: Was ist neu? Vergleich der aktuellen und bisherigen Empfehlungen zu Diagnostik und Chirurgie

**DOI:** 10.1007/s00106-025-01654-9

**Published:** 2025-09-01

**Authors:** Lukas Kuczera, Sarina Katrin Müller, Matti Sievert

**Affiliations:** Hals-Nasen-Ohren-Klinik, Kopf- und Halschirurgie, Universitätsklinikum Erlangen, Waldstraße 1, 91054 Erlangen, Deutschland

**Keywords:** Schilddrüsenknoten, Drüsige und epitheliale Neoplasien, Diagnostik, Therapie, Leitlinien, Thyroid nodule, Glandular and epithelial neoplasms, Diagnostics, Treatment, Guidelines

## Abstract

**Hintergrund:**

Aufgrund ihrer hohen Prävalenz erfordert der Umgang mit Schilddrüsenknoten in besonderem Maße ein systematisches Vorgehen. Ergibt die Diagnostik den Verdacht oder Nachweis eines Schilddrüsenkarzinoms, bleibt die chirurgische Therapie nach wie vor in den meisten Fällen die vordergründige Behandlungsoption.

**Fragestellung:**

Ziel dieser Arbeit ist es, einen Überblick über das leitliniengerechte Vorgehen bei Schilddrüsenknoten und -karzinomen mit Fokus auf Diagnostik und chirurgische Therapie zu geben. Hierbei werden wesentliche inhaltliche Unterschiede zwischen der bisherigen und der aktuellen Leitlinie herausgearbeitet.

**Material und Methoden:**

Selektion der praxisrelevantesten Empfehlungen der 2025 erschienenen Leitlinie „Schilddrüsenkarzinom“ und Vergleich dieser mit den Empfehlungen der 2012 erschienenen Leitlinie „Operative Therapie maligner Schilddrüsenerkrankungen“.

**Ergebnisse:**

Die neue Leitlinie ist deutlich umfangreicher und detaillierter. Das empfohlene diagnostische Vorgehen wird präzisiert und durch Untersuchungstechniken wie die Elastographie ergänzt. Das operative Vorgehen behält seine Rolle als primäre therapeutische Säule. Entsprechend der aktuellen Studienlage werden hier neue Optionen, wie das beobachtende Verhalten papillärer Mikrokarzinome unter definierten Voraussetzungen, aufgenommen. Die Empfehlungen zu Diagnostik und Therapie undifferenzierter Schilddrüsenkarzinome wurden maßgeblich erweitert.

**Schlussfolgerung:**

Die neue Leitlinie gibt einen umfassenden Überblick über Diagnostik und Therapie bei Schilddrüsenkarzinomen und erweitert die bisherige Leitlinie unter Berücksichtigung der aktuellen Studienlage.

## Abstract (en)

**Background:**

Due to their high prevalence the management of thyroid nodules requires a particularly systematic approach. If the diagnostics reveal a suspected or confirmed thyroid cancer, in most cases surgical treatment remains the primary treatment option.

**Objective:**

The aim of this work is to provide an overview of the guideline-based approach to thyroid nodules and carcinomas with a focus on diagnostics and surgical treatment. The essential differences in content between the previous and current guidelines are identified.

**Material and methods:**

Selection of the most practice-relevant recommendations of the guidelines on thyroid cancer published in 2025 and comparison of these with the recommendations of the guidelines on surgical treatment of malignant thyroid diseases published in 2012.

**Results:**

The new guidelines are significantly more comprehensive and detailed. The recommended diagnostic procedure is more precise and supplemented by examination techniques such as elastography. The surgical procedure keeps its role as the primary treatment approach. In accordance with the current study situation new options, such as the observational behavior for papillary microcarcinomas under defined conditions, are included. The recommendations on the diagnostics and treatment of undifferentiated thyroid cancer have been substantially expanded.

**Conclusion:**

The new guidelines provide a comprehensive overview of the diagnostics and treatment of thyroid cancer and expands on the previous guidelines with consideration of the current study situation.

Schilddrüsenknoten sind ein häufiges Thema im klinischen Alltag. Je nach Studie lässt sich bei etwa jedem 20. Patienten bereits durch Palpation ein auffälliger Schilddrüsenbefund feststellen [1]. Mithilfe der Sonographie zeigen sich in Deutschland Prävalenzen von etwa 20 % [2]. Obwohl nur etwa 1 % der entdeckten Knoten tatsächlich maligne sind, erfordert die hohe Anzahl von Fällen ein differenziertes und strukturiertes diagnostisches und therapeutisches Vorgehen [3].

Die neue Leitlinie „Schilddrüsenkarzinom“ bildet die Grundlage für ein solches Vorgehen im Hinblick auf die verschiedenen Tumorentitäten. Im Vergleich zur 2012 erschienenen Leitlinie „Operative Therapie maligner Schilddrüsenerkrankungen“ präsentiert sie sich deutlich detaillierter und umfangreicher. Der vorliegende Beitrag gibt einen Überblick über die zentralen Gemeinsamkeiten und Unterschiede zwischen den beiden Leitlinien, mit einem besonderen Fokus auf die Diagnostik und die operative Therapie (Tab. 1) von Schilddrüsenknoten und Schilddrüsenkarzinomen.

**Tab. 1:** Übersicht über die Wahl des Ausmaßes der Resektion des Primärtumors bei Schilddrüsenkarzinomen nach histologischem Subtyp: Es handelt sich um einen deutlich vereinfachten Überblick ohne Anspruch auf Vollständigkeit. Die spezifischen Ausführungen der Leitlinie sowie die Indikationen zur Lymphknotendissektion sind zu beachten

	Hemithyreoidektomie	Totale Thyreoidektomie	Anmerkungen
*Papilläres Schilddrüsenkarzinom (PTC)*			
Befunde > 1 cm	(●) begründete Fälle ohne Risikofaktoren	●	–
Kapselüberschreitendes Wachstum, Metastasierung, multifokales Wachstum, familiäre PTC, aggressive histologische Varianten	–	●	–
*Papilläres Schilddrüsenmikrokarzinom (≤* *1* *cm)*			
Isoliert, unifokal, ohne Risikofaktoren	●	–	Beobachtendes Verhalten bei Patienten > 30 LJ nach ausführlicher Aufklärung möglich, lokal-ablative Verfahren nur in Einzelfällen nach Tumorboard
Mit Risikofaktoren	–	●	–
*Follikuläres Schilddrüsenkarzinom*			
Minimal-invasiv, Primärtumor ≥ 4 cm	–	●	–
Minimal-invasiv, Primärtumor < 4 cm	●	–	–
Bei Angioinvasion, jede Tumorgröße	–	●	–
Breit-invasiv	–	●	–
*Onkozytäres Schilddrüsenkarzinom*	–	●	–
*Gering differenziertes Schilddrüsenkarzinom*	–	●	Bei organüberschreitenden Befunden unter lokal-kurativer Intention: Resektion am Aerodigestivtrakt
*Medulläres Schilddrüsenkarzinom*	–	●	Keine präoperative Feinnadelpunktion, Ausmaß der Lymphknotendissektion u. a. abhängig vom Calcitoninwert
*Anaplastisches Schilddrüsenkarzinom*	–	●	Ggf. neoadjuvante Konzepte, Relevanz der nichtoperativen Therapie beachten
Unilateral, intrathyroidal	(●) Erwägen, falls vollständige Resektion möglich	–	–

## Diagnostik

Eine ausführliche Anamnese mit Familienanamnese und die Erhebung eines klinischen Untersuchungsbefunds, insbesondere mit Palpation der Schilddrüsenregion, stehen weiterhin am Anfang jeder Diagnostik. Eine zusätzliche Strahlenanamnese sollte erhoben werden, da insbesondere eine Bestrahlung des Halses in der Kindheit als Risikofaktor angesehen wird. Im Rahmen der HNO-ärztlichen Vorstellung sollen vordergründig auch subjektive Stimm‑, Atem- und Schluckbeschwerden erfasst werden, und es soll eine Laryngoskopie zur Beurteilung der respiratorischen Beweglichkeit erfolgen.

### Sonographie

Die Sonographie spielt nach wie vor eine zentrale Rolle in der Diagnostik von Schilddrüsenerkrankungen. Der geforderte Umfang und Ablauf dieser Untersuchung wurden nun in der aktuellen Leitlinie präzisiert. Während die bisherige Leitlinie die Sonographie als essenzielle präoperative Untersuchungsmethode zur Beurteilung der Schilddrüse und extrathyroidaler Strukturen empfahl, erweitert die aktualisierte Leitlinie diesen Ansatz. Sie fordert eine standardisierte und strukturierte Bewertung von Schilddrüsenknoten im Rahmen der B‑Mode-Sonographie unter Anwendung eines TI-RADS(Thyroid Imaging Reporting and Data System)-Klassifikationssystems, um die diagnostische Präzision zu erhöhen und eine reproduzierbare Risikostratifizierung zu gewährleisten. Zwar spricht die Leitlinie keine explizite Empfehlung für ein spezifisches Klassifikationssystem aus, jedoch wird ein leichter Vorteil von Kwak-TI-RADS, ACR(American College of Radiology)-TI-RADS und K(koreanisches)-TI-RADS gegenüber EU(europäisches)-TI-RADS hervorgehoben [4].

Ergänzend zur B‑Mode-Sonographie, wird, sofern verfügbar, die Durchführung einer Elastographie zur Untersuchung von Schilddrüsenknoten empfohlen. Besonders hervorgehoben wird hierbei der hohe negative prädiktive Wert eines weichen Knotens [5, 6]. Die farbkodierte Duplexsonographie (FKDS) hingegen wird in Bezug auf die Bewertung des Malignitätsrisikos von Schilddrüsenknoten als entbehrlich angesehen. In Bezug auf die kontrastverstärkte Ultrasonographie („contrast-enhanced ultrasound“, CEUS) wird auf eine fehlende Standardisierung hingewiesen und aufgrund der unzureichenden Studienlage aktuell keine Empfehlung ausgesprochen.

Zur Beurteilung möglicher Lymphknotenmetastasen bleibt die systematische sonographische Untersuchung der Lymphabflussgebiete der Schilddrüsenregion bei Malignitätsverdacht weiterhin unerlässlich. Die Einordnung der untersuchten Lymphknoten anhand evaluierter sonomorphologischer Kriterien und unter Zuhilfenahme der FKDS ist sinnvoll. Aufgrund der noch unzureichenden Datenlage wird derzeit keine Empfehlung zur Anwendung der Elastographie zur Dignitätsbeurteilung von Lymphknoten bei suspekten Schilddrüsenknoten ausgesprochen.

### Szintigraphie

Die szintigraphische Beurteilung der Schilddrüse mittels Technetium(Tc)-99m-Pertechnetat- oder Jod-123-Szintigraphie bleibt weiterhin ein zentraler Bestandteil der diagnostischen Abklärung. Die neue Leitlinie nimmt in diesem Bereich jedoch eine differenziertere Position ein: Ab einer festgelegten Knotengröße von mindestens 1 cm soll eine Szintigraphie zur Dignitätseinschätzung nun unabhängig vom TSH(thyreoideastimulierendes Hormon)-Wert erfolgen. Hyperfunktionelle Knoten sollen im Regelfall explizit nicht weiter abgeklärt werden. Diese Empfehlung basiert auf der Analyse von 65 Publikationen über initial hyperfunktionell imponierende Schilddrüsenmalignome, von denen lediglich 10 Publikationen einer genaueren Überprüfung standhielten [7]. Ein hypofunktioneller Knoten allein stellt hingegen, unter Berücksichtigung der niedrigen Malignomprävalenzen in Studien [8], keine ausreichende Indikation zur Operation dar. Vielmehr soll die Szintigraphie im Kontext weiterer diagnostischer Verfahren betrachtet werden, um ausreichend hohe Vorhersagewerte zu erreichen, die ein operatives Vorgehen rechtfertigen. Bezüglich der Bewertung indifferenter Knoten kann aufgrund fehlender belastbarer Daten keine klare Empfehlung ausgesprochen werden. Für diese Fälle wird eine Suppressionsszintigraphie als Verfahren der Wahl vorgeschlagen, um eine mögliche Hyperfunktionalität besser darstellbar zu machen.

### Laborchemische Diagnostik

Für die laborchemische Diagnostik wird nun die Bestimmung des TSH-Wertes im Rahmen der Abklärung von Schilddrüsenknoten standardmäßig empfohlen. Erniedrigte TSH-Werte können dabei auf eine Autonomie hinweisen [9]. Gleichzeitig wird auf die heterogene Studienlage verwiesen, die sowohl bei erhöhten als auch bei erniedrigten TSH-Werten ein erhöhtes Karzinomrisiko nahelegt [10]. Weiterhin spielt die Bestimmung des Thyreoglobulinwertes in der Primärdiagnostik keine relevante Rolle, kommt jedoch in der Nachsorge zum Einsatz. Die neue Leitlinie geht zudem auf die Bestimmung von Thyreoglobulin- (TAK) und Thyreoperoxidaseantikörpern (TPO-AK) ein. Studien zeigen, dass positive TAK mit einem höheren Risiko für ein papilläres Schilddrüsenkarzinom assoziiert sein könnten und möglicherweise auf ein höheres Tumorstadium hindeuten. Auch für TPO-AK deutet die Studienlage auf ein erhöhtes Risiko für das Vorhandensein differenzierter Schilddrüsenkarzinome hin [11]. Aus diesen Daten wird jedoch keine evidenzbasierte Empfehlung abgeleitet. Die Bestimmung des basalen Calcitonins als Marker für das medulläre Schilddrüsenkarzinom sollte weiterhin routinemäßig mittels Doppelantikörper-Immunoassay erfolgen.

### Diagnostische Punktion

Die Feinnadelpunktion von Schilddrüsenknoten ist weiterhin nur bei sonographisch suspekten Knoten indiziert und sollte grundsätzlich ultraschallgesteuert erfolgen. Neu ist die explizite Empfehlung, nur noch Knoten mit einer Mindestgröße von 1 cm zu punktieren. Für Knoten mit einem Durchmesser unter 1 cm wird die Punktion anstelle sonographischer Kontrollen nur noch bei Vorliegen zusätzlicher Risikofaktoren und bei Vorhandensein der entsprechenden Expertise der Punktierenden empfohlen.

Ebenfalls neu ist die Empfehlung, bei Verdacht auf medulläre Schilddrüsenkarzinome keine Feinnadelpunktion mehr durchzuführen. Begründet wird dies mit dem Hinweis auf die mögliche Auslösung einer fibroblastären Proliferation, die einer intraoperativen Gefrierschnittdiagnostik entgegenstehen kann [12]. Die Gewebereaktion durch die Biopsie gleiche hierbei mikroskopisch einer desmoplastischen Stromareaktion, welche wiederum ein invasives Tumorwachstum anzeigen kann. Tumoren ohne solche Stromareaktion bilden in der Regel keine Lymphknotenmetastasen.

Die Grobnadelbiopsie hat aufgrund der fehlenden Vorteile gegenüber der Feinnadelbiopsie in der Primärdiagnostik weiterhin einen untergeordneten Stellenwert. Beschriebene Vorteile bei nichtdiagnostischen Feinnadelpunktionen und insbesondere bei Knoten mit Makroverkalkungen und starken regressiven Veränderungen werden jedoch zitiert [13]. Ausnahmen stellen der Verdacht auf ein anaplastisches Schilddrüsenkarzinom oder Lymphome dar.

Zur Einordnung der Feinnadelbiopsie soll ein etabliertes zytologisches Befundungssystem verwendet werden. Vor dem Hintergrund der vorgeschlagenen strikten Punktionsindikation werden hier 5‑stufige Befundungssysteme, entgegen der 6‑stufigen Bethesda-Klassifikation von der American Thyroid Association (ATA), im Sinne einer „Kann-Empfehlung“ vorgeschlagen. Hierdurch sollen sonst als Bethesda III (follikuläre Läsion unklarer Signifikanz/Atypie unklarer Signifikanz) eingestufte zytologische Präparate einem aussagekräftigeren Grad zugeordnet werden.

### Weitere bildgebende Verfahren

In der bisherigen Leitlinie wurde bei Notwendigkeit zur weiteren Einschätzung der Lokalausdehnung sowie bei V. a. ausgeprägte Lymphknotenmetastasierung die Magnetresonanztomographie (MRT) als bevorzugte Schnittbildgebung vorgeschlagen. Dies kann weiterhin so beibehalten werden. Ebenso sollte weiterhin die Gabe von jodhaltigem Kontrastmittel bei V. a. Schilddrüsenkarzinom vermieden werden; sie ist dem Ausnahmefall vorbehalten. Die Computertomographie (CT) sollte insgesamt nicht als initiales Routine-Staging angewendet werden. Eine Ausnahme bildet hier die Diagnostik möglicher Lungenmetastasen mittels Nativ-CT. Ist ein differenziertes Schilddrüsenkarzinom ausgeschlossen, wird demgegenüber bei fortgeschrittenem Tumorleiden die kontrastmittelgestützte CT zum Staging explizit empfohlen, falls verfügbar, mittels 18F-FDG(Fluordesoxyglukose)-PET (Positronenemissionstomographie). Bei gegebener Indikation zur Feinnadelpunktion kann die Bildgebung mit ^99m^Tc-MIBI (Methoxy-iso-butyl-isonitril) als Alternative genutzt werden, wenn eine Feinnadelpunktion nicht durchführbar ist, vom Patienten abgelehnt wird, kein repräsentatives Material liefert oder zu keinem aussagekräftigen Befund führt. Insbesondere der hohe negativ prädiktive Wert dieser Methode ist hierbei hervorzuheben. Ein generelles Screening mit ^99m^Tc-MIBI wird nicht empfohlen.

## Operative Therapie

Entsprechend den diagnostischen Handlungsempfehlung ist die neue Leitlinie auch bezüglich der therapeutischen Empfehlungen präziser geworden. Zur Gewährleistung einer optimalen individuellen Versorgung nach aktuellem Kenntnisstand ist die Empfehlung zur regelhaften Fallvorstellung in einem interdisziplinären Tumorboard bei Verdacht auf Schilddrüsenkarzinom schriftlich in der Leitlinie fixiert worden. Zur Schaffung einer einheitlichen Terminologie sind nun ebenso die Resektionsformen der Schilddrüse in der Leitlinie definiert. Für lokoregionäre Lymphknotenresektionen wird weiterhin die Kompartmentklassifikation [14] entgegen der international gängigen Robbins-Klassifikation angeführt [15].

### Papilläres Schilddrüsenkarzinom

Beim häufigsten malignen Schilddrüsenkarzinom, dem papillären Schilddrüsenkarzinom (PTC), ist die Thyreoidektomie wie bisher bei Befunden größer als 1 cm sowie größenunabhängig bei kapselüberschreitendem Wachstum und Metastasierung empfohlen. Hinzugefügt wurde die explizite Empfehlung zur totalen Thyreoidektomie auch bei multifokalen Befunden, familiären PTC und aggressiveren histologischen Varianten (z. B. Columnar-Zell- oder Hobnail-Variante). In Anlehnung an die ATA-Empfehlung, die eine totale Thyreoidektomie bei Low-risk-Karzinomen erst ab einer Tumorgröße von über 4 cm oder bei Vorliegen zusätzlicher Risikofaktoren empfiehlt, kann in begründeten Fällen und bei Fehlen von Risikofaktoren bei pT1b/pT2-Befunden auch eine Hemithyreoidektomie in Betracht gezogen werden. Es wird jedoch die divergierende Studienlage bezüglich des resultierenden Gesamtüberlebens und der Rezidivrate thematisiert. Studienergebnisse, welche die Lobektomie nach Empfehlungen der ATA begründen, werden hierbei deutlich kritisch hinterfragt [16]. Eine fundierte Patientenaufklärung im Sinne eines „informed consent“ ist hierbei essenziell.

Unverändert bleiben die Empfehlungen zur zentralen und lateralen Kompartmentknotendissektion. Bei cN0-Befunden mit einem Primarius größer als 1 cm sollte eine prophylaktische zentrale Kompartmentresektion (nur begründet und nur bei entsprechender operativer Expertise) erfolgen. Es werden diesbezüglich die Vorteile (exaktes histopathologisches Staging, Entfernung häufiger Mikrometastasen, geringere Rezidivrate, höhere Wahrscheinlichkeit der postoperativen Normalisierung von Thyreoglobulin) gegenüber den Nachteilen (kein gesicherter onkologischer Vorteil; höheres Komplikationsrisiko, insbesondere in Bezug auf den postoperativen Hypoparathyreoidismus) letztlich zugunsten der prophylaktischen zentralen Kompartmentdissektion breit diskutiert [17, 18]. Bei postoperativer Diagnose eines PTC ohne Hinweis auf einen Resttumor und bestehendem cN0-Halsstatus ist eine komplettierende prophylaktische zentrale Kompartmentdissektion weiterhin nicht empfohlen. Bei jedem PTC mit cN1-Halsstatus ist die zentrale Kompartmentresektion wie bisher indiziert. Bei Lokalisation des PTC im oberen Pol oder ausgedehntem zentralen Lymphknotenbefall und bei klinischem Verdacht bzw. Nachweis von lateralen Lymphknotenmetastasen ist die laterale Kompartmentresektion der tumortragenden Seite weiterhin empfohlen. Bei ausgedehntem zentralen Befall sollte ggf. auch die laterale Kompartmentresektion der kontralateralen Seite durchgeführt werden. Angeführt wird hier eine Studie, in der bei mehr als 5 zentralen Lymphknotenmetastasen bei der Mehrzahl der Patienten sowohl das ipsilaterale als auch das kontralaterale Kompartiment als befallen beschrieben werden konnte [19]. Ohne Hinweis auf laterale Lymphknotenmetastasen soll grundsätzlich auch weiterhin keine prophylaktische laterale Kompartmentresektion erfolgen. Wie auch bisher sollte eine Komplettierungsoperation immer dann stattfinden, wenn die Primäroperation nicht dem ursprünglich angemessenen Resektionsverfahren entsprochen hat.

### Papilläres Schilddrüsenmikrokarzinom

Bezüglich der Therapieoptionen bei papillären Schilddrüsenmikrokarzinomen (PTMC), also papillären Karzinomen bis maximal 1 cm Durchmesser, ist die neue Leitlinie ebenfalls detaillierter geworden. Neu ist hierbei die nun festgelegte Möglichkeit, isolierte unifokale PTMC ohne Risikofaktoren (insbesondere multifokales Wachstum, familiäre Häufung, Lymphknotenmetastasen, risikoreiche histologische Subtypen, vorausgegangene zervikale Bestrahlung, fehlende Kapselbegrenzung oder Lage an der Trachea bzw. im Bereich des Nervus laryngeus recurrens) ausschließlich zu beobachten. Voraussetzung ist, vor dem Hintergrund der Wahrscheinlichkeiten einer Erkrankungsprogression auf die Lebenszeit, ein Patientenalter über 30 Jahre. In der hervorgehobenen Studie betrugen diese für einen Beobachtungszeitraum von 10 Jahren exemplarisch 60,3 % für 20- bis 29-Jährige und 6,6 % für die Gruppe der 50- bis 59-Jährigen [20]. Voraussetzung für dieses Prozedere ist ebenfalls eine ausführliche Informierung des Patienten im Sine eines „informed consent“.

Neu aufgefasst und kritisch diskutiert wird die Möglichkeit der lokal-ablativen Verfahren. Die beschriebene Effektivität und Komplikationsarmut werden zwar angeführt [21], jedoch werden das Fehlen prospektiver Vergleichsstudien zu den klassischen chirurgischen Vorgehensweisen und teilweise die Qualität der bisherigen Studien bemängelt. Dementsprechend wird keine evidenzbasierte Empfehlung abgegeben. Es ist zwingend erforderlich, den Patienten über die Bedeutung der fehlenden histologischen Sicherung sowie über mögliche Einschränkungen der sonographischen Verlaufsbeurteilung aufzuklären und diese Informationen einer Aufklärung über die etablierte operative Therapie gegenüberzustellen. Die Entscheidung für eine lokal-ablative Therapie soll durch ein interdisziplinäres Tumorboard getroffen und idealerweise nur im Rahmen einer Studienaufnahme durchgeführt werden.

Bei Entscheidung von Patienten und Therapeut für eine operative Therapie des PTMC ist das Vorgehen nun prägnanter als bisher festgelegt. Für PTMC ohne Risikofaktoren (siehe oben) sollte die Hemithyreoidektomie, für PTMC mit Risikofaktoren (also auch für multifokale PTMC) die totale Thyreoidektomie erfolgen. Es wird jedoch wie bisher darauf hingewiesen, dass der Schwellenwert von 1 cm Durchmesser tumorbiologisch ein Kontinuum darstellt und daher im Grenz- und Einzelfall interdisziplinär und individuell entschieden werden sollte. Eine prophylaktische zentrale Kompartmentresektion kann bei Vorliegen von Risikofaktoren, zu denen nun explizit auch eine Größe ab etwa 8–10 mm gezählt wird, durchgeführt werden. Auf cN1-PTMC wurde in der bisherigen Leitlinie nicht gesondert eingegangen. Hier ist die totale Thyreoidektomie mit bilateraler zentraler Kompartmentresektion empfohlen.

### Follikuläres Schilddrüsenkarzinom

Bei follikulären Schilddrüsenkarzinomen muss aufgrund ihrer Tumorbiologie und Prognose weiterhin grundsätzlich das minimal-invasive follikuläre Karzinom mit bzw. ohne Angioinvasion vom breit-invasiven follikulären Schilddrüsenkarzinom unterschieden werden. Für solitäre minimal-invasive follikuläre Schilddrüsenkarzinome ohne Kapselinvasion und ohne Angioinvasion war bisher unabhängig von der Tumorgröße die Durchführung einer primären oder sekundären totalen Thyreoidektomie nicht erforderlich. In der nun geltenden Leitlinie wird für große Primärtumoren ab 4 cm ohne histologisch nachweisbare Angioinvasion die totale Thyreoidektomie empfohlen. Bei Tumorgröße unter 4 cm ist weiterhin die Hemithyreoidektomie vorzuziehen. Bei histopathologischer Angioinvasion sollte weiterhin unabhängig von der Tumorgröße die totale Thyreoidektomie erfolgen. Neu ist die Kann-Empfehlung, eine prophylaktische Lymphknotendissektion bei minimal-invasiven follikulären Schilddrüsenkarzinomen mit Kapselinvasion in Erwägung zu ziehen. Abgesehen davon, ist die prophylaktische Lymphknotendissektion bei diesem Subtyp, unter Hinweis auf die niedrigen lymphogenen Metastasierungsraten von weniger als 10 % [22], nicht empfohlen.

Insbesondere aufgrund des hohen Fernmetastasierungsrisikos [22] unterscheidet sich das therapeutische Vorgehen beim breit-invasiven follikulären Karzinom. Es sollte hier weiterhin die totale Thyreoidektomie erfolgen, ggf. auch im Sinne einer Komplettierungsthyreoidektomie bei erst postoperativer Diagnose. Unverändert bleibt die Empfehlung zur zentralen Kompartmentresektion bei prä- oder intraoperativ nachgewiesener Lymphknotenmetastasierung.

### Medulläres Schilddrüsenkarzinom

Die Sonderstellung des medullären Schilddrüsenkarzinoms als ein von den hormonproduzierenden C‑Zellen ausgehender Tumor bedingt damit einhergehende Besonderheiten im diagnostischen und therapeutischen Vorgehen. Wenngleich die allgemeine diagnostische Vorgehensweise bei Schilddrüsenknoten auch für das medulläre Schilddrüsenkarzinom Anwendung finden soll, wird das Prüfen spezifischer anamnestischer und klinischer Hinweise auf diese Entität nun als explizite Handlungsempfehlung aufgenommen. Hierzu zählen insbesondere erhöhte Calcitonin- und CEA(karzinoembryonales Antigen)-Spiegel, therapieresistente Durchfälle sowie eine positive Eigen- oder Familienanamnese für Phäochromozytome oder primären Hyperparathyreoidismus als Hinweis auf eine multiple endokrine Neoplasie Typ 2. Der Calcitoninwert behält hierbei seine wichtige Stellung als Tumormarker unter Berücksichtigung einiger Anpassungen. So sollen Stimulationstests für Calcitonin nun nur noch in Einzelfällen erfolgen. Grundsätzlich soll der Calcitoninwert zumindest präoperativ und bei sonographisch suspekten Befunden bestimmt werden. Der Grenzwert, ab welchem mit hoher Wahrscheinlichkeit ein medulläres Schilddrüsenkarzinom vorliegt, wird hierbei nun auf 30 pg/ml für Frauen und 60 pg/ml für Männer festgelegt (zuvor: geschlechtsunabhängig stimulierte Calcitoninwerte über 100 pg/ml). Bei gleichzeitigem Nachweis eines Schilddrüsenknotens ist mit diesen Werten die operative Therapie bereits gerechtfertigt und empfohlen. CEA als zweiter Tumormarker soll nicht zur präoperativen Diagnostik eingesetzt werden, stellt aber weiterhin einen stabilen Marker zum Tumormonitoring und in der Verlaufskontrolle des metastasierten medullären Schilddrüsenkarzinoms dar.

Die Empfehlungen zum Ausmaß der Resektion sind unter Berücksichtigung der neuen Grenzwerte größtenteils unverändert geblieben. Aufgrund des relativ häufigen multifokalen Auftretens ist die totale Thyreoidektomie empfohlen. Vor dem Hintergrund der häufig frühzeitigen lokalen Metastasierung soll die beidseitige zentrale Kompartmentdissektion im Rahmen des Ersteingriffs erfolgen. Die einseitige laterale Kompartmentdissektion auf der tumortragenden Seite soll bestenfalls ebenso im Ersteingriff erfolgen, kann im Einzelfall aber auch im Rahmen einer Zweitoperation durchgeführt werden, wenn der Calcitoninwert 6 bis 12 Wochen postoperativ nicht unterhalb der Nachweisgrenze liegt. Eine weitere Ausnahme stellen medulläre Schilddrüsenkarzinome bis maximal 1 cm Durchmesser dar, die gleichzeitig einen Calcitoninwert unterhalb des Grenzwertes zeigten. Auch hier kann aufgrund der geringen Metastasierungsrate auf die laterale Kompartmentresektion im Ersteingriff verzichtet werden. Spätestens ab einem basalen Calcitoninwert von mehr als 200 pg/ml soll die kontralaterale Kompartmentdissektion erfolgen. Bei fehlender desmoplastischer Stromareaktion im Gefrierschnitt sollte auf eine einzeitige zervikale Lymphknotendissektion verzichtet werden.

### Anaplastische Schilddrüsenkarzinome

Das anaplastische Schilddrüsenkarzinom stellt einen onkologischen Notfall dar, der eine sofortige und gezielte Therapie erfordert. Aufgrund der aggressiven Natur der Erkrankung muss die Behandlungsstrategie stets als Einzelfallentscheidung erfolgen und in einem spezialisierten, interdisziplinären Tumorboard diskutiert werden. Zudem ist die Studienlage zu neuen Therapieansätzen einem stetigen Wandel unterworfen, weshalb aktuelle klinische Studien eine zunehmend wichtige Rolle in der Behandlung spielen.

Die aktuelle Leitlinie enthält hierzu deutlich detailliertere Empfehlungen als ihre Vorgängerversion. So wird neben einer geeigneten Bildgebung zur Erfassung der lokalen Tumorausbreitung nun explizit eine Ganzkörper-PET/CT mit 18F-FDG empfohlen. Zudem sollen bereits bei Diagnosestellung, beispielsweise durch Stanzbiopsie, eine molekularpathologische Panelsequenzierung und die immunhistochemische Bestimmung der PD-L1(„programmed death ligand 1“)-Expression erfolgen. Bezüglich der operativen Therapie bleibt eine radikale Resektion das Ziel, sofern eine R0(/ggf. R1-)-Resektion realistisch erscheint. Während unilaterale und intrathyroidal begrenzte Tumoren weiterhin in Einzelfällen mittels Hemithyreoidektomie ausreichend reseziert werden können, bleibt in den meisten Fällen eine totale Thyreoidektomie erforderlich. Die zervikozentrale Kompartmentdissektion wird uneinheitlich bewertet, ohne dass eine klare Handlungsempfehlung gegeben wird. Eine laterale Kompartmentdissektion ist – wie bereits in der bisherigen Leitlinie – nur bei klinischem Befall zu erwägen. Neu aufgenommen wurde das neoadjuvante Konzept für lokal begrenztes extrathyroidales Tumorwachstum mit dem Ziel einer nachfolgenden operativen Resektion. Bei nichtresektablen Tumoren mit *BRAF*(„B-rapidly accelerated fibrosarcoma“)-V600E-Mutation wird eine neoadjuvante Therapie empfohlen. In einer Studie erreichten 70 % der Patienten nach mindestens 1‑monatiger Behandlung mit Dabrafenib und Trametinib eine Resektabilität. Zudem reduzierte die neoadjuvante Therapie das Operationsausmaß und die damit verbundene Morbidität [23]. Dementsprechend sollte die operative Therapie immer dann an eine neoadjuvante Behandlung anschließen, wenn dadurch eine Resektabilität erzielt werden kann. Darüber hinaus wird auf die Bedeutung adjuvanter und primär nichtoperativer Therapieoptionen, insbesondere Radio- und Systemtherapien, hingewiesen. Die Aufnahme von zielgerichteten Therapien in die aktuelle Leitlinie erweitert das therapeutische Spektrum erheblich; sie stellen neben der operativen Therapie eine weitere zentrale Behandlungsoption dar. Im Zuge der rasanten Entwicklungen in der klinischen Forschung werden zunehmend neue Therapieansätze im Rahmen von Studien erprobt, die zukünftig die Behandlungsmöglichkeiten verbessern könnten.

## Fazit für die Praxis

- Anamnese, klinische Untersuchung, Sonographie und Laryngoskopie stellen die Basis der Schilddrüsendiagnostik dar.
- Schilddrüsenknoten sollten sonographisch anhand einer TI-RADS(Thyroid Imaging Reporting and Data System)-Klassifikation bewertet werden. Die Durchführung einer Elastographie ist empfohlen.
- Schilddrüsenknoten größer 1 cm bedürfen der weiteren Abklärung mittels Szintigraphie.
- Die Bestimmung des TSH(thyreoideastimulierendes Hormon)- und des Calcitoninwertes ist standardmäßig empfohlen.
- Suspekt eingeschätzte Schilddrüsenknoten größer 1 cm sollen feinnadelpunktiert werden. Besteht der Verdacht eines medullären Schilddrüsenkarzinoms, soll keine Punktion erfolgen.
- Die operative Therapie stellt i. d. R. bei allen Formen des Schilddrüsenkarzinoms das primäre Vorgehen der Wahl dar.
- Bei papillären Schilddrüsenkarzinomen größer 1 cm ist die Thyreoidektomie weiterhin indiziert. In Ausnahmefällen darf die Hemithyreoidektomie diskutiert werden.
- Bei unifokalen papillären Mikrokarzinomen ohne Risikofaktoren soll die Hemithyreoidektomie erfolgen. Unter strengen Voraussetzungen darf der Befund nun auch beobachtet werden.

## References

[1] Dean DS, Gharib H (2008) Epidemiology of thyroid nodules. Best Pract Res Clin Endocrinol Metab 22(6):901–911 19041821 10.1016/j.beem.2008.09.019

[2] Völzke H, Lüdemann J, Robinson DM, Spieker KW, Schwahn C, Kramer A, John U, Meng W (2003) The prevalence of undiagnosed thyroid disorders in a previously iodine-deficient area. Thyroid 13(8):803–810 14558922 10.1089/105072503768499680

[3] Grussendorf M, Ruschenburg I, Brabant G (2022) Malignancy rates in thyroid nodules: a long-term cohort study of 17,592 patients. Eur Thyroid J 11(4):e220027 35635802 10.1530/ETJ-22-0027 PMC9254276

[4] Seifert P, Schenke S, Zimny M, Stahl A, Grunert M, Klemenz B, Freesmeyer M, Kreissl MC, Herrmann K, Görges R (2021) Diagnostic Performance of Kwak, EU, ACR, and Korean TIRADS as Well as ATA Guidelines for the Ultrasound Risk Stratification of Non-Autonomously Functioning Thyroid Nodules in a Region with Long History of Iodine Deficiency: A German Multicenter Trial. Cancers 13(17):4467 34503277 10.3390/cancers13174467 PMC8431215

[5] Russ G, Bonnema SJ, Erdogan MF, Durante C, Ngu R, Leenhardt L (2017) European Thyroid Association Guidelines for Ultrasound Malignancy Risk Stratification of Thyroid Nodules in Adults: The EU-TIRADS. Eur Thyroid J 6(5):225–237 29167761 10.1159/000478927 PMC5652895

[6] Nell S, Kist JW, Debray TP, de Keizer B, van Oostenbrugge TJ, Borel Rinkes IH, Valk GD, Vriens MR (2015) Qualitative elastography can replace thyroid nodule fine-needle aspiration in patients with soft thyroid nodules. A systematic review and meta-analysis. Eur J Radiol 84(4):652–661 25638577 10.1016/j.ejrad.2015.01.003

[7] Schröder S, Marthaler B (1996) Autonomie und Malignität bei Schilddrüsentumoren. Eine kritische Literaturanalyse zur Existenz hyperfunktionierender follikulärer und papillärer Schilddrüsenkarzinome [Autonomy and malignancy of thyroid glad tumors. A critical analysis of the literature on the existence of hyperfunctioning follicular and papillary thyroid gland carcinomas. Pathologe 17(5):349–357 8992477 10.1007/s002920050172

[8] Powers AE, Marcadis AR, Lee M, Morris LGT, Marti JL (2019) Changes in Trends in Thyroid Cancer Incidence in the United States, 1992 to 2016. JAMA 322(24):2440–2441 31860035 10.1001/jama.2019.18528 PMC6990659

[9] Biondi B, Bartalena L, Cooper DS, Hegedüs L, Laurberg P, Kahaly GJ (2015) The 2015 European Thyroid Association Guidelines on Diagnosis and Treatment of Endogenous Subclinical Hyperthyroidism. Eur Thyroid J 4(3):149–163 26558232 10.1159/000438750 PMC4637513

[10] Schiffmann L, Kostev K, Kalder M (2020) Association between various thyroid gland diseases, TSH values and thyroid cancer: a case-control study. J Cancer Res Clin Oncol 146(11):2989–2994 32518973 10.1007/s00432-020-03283-x PMC11804546

[11] Zhao H, Li H, Huang T (2018) High Urinary Iodine, Thyroid Autoantibodies, and Thyroid-Stimulating Hormone for Papillary Thyroid Cancer Risk. Biol Trace Elem Res 184(2):317–324 29164514 10.1007/s12011-017-1209-6

[12] Scheuba C, Kaserer K, Kaczirek K, Asari R, Niederle B (2006) Desmoplastic stromal reaction in medullary thyroid cancer—an intraoperative “marker” for lymph node metastases. World j surg 30(5):853–859 16680600 10.1007/s00268-005-0391-4

[13] Na DG, Kim DS, Kim SJ, Ryoo JW, Jung SL (2016) Thyroid nodules with isolated macrocalcification: malignancy risk and diagnostic efficacy of fine-needle aspiration and core needle biopsy. Ultrasonography 35(3):212–219 26810196 10.14366/usg.15074 PMC4939718

[14] Dralle H, Damm I, Scheumann GF, Kotzerke J, Kupsch E, Geerlings H, Pichlmayr R (1994) Compartment-oriented microdissection of regional lymph nodes in medullary thyroid carcinoma. Surg Today 24(2):112–121 8054788 10.1007/BF02473391

[15] Robbins KT, Shaha AR, Medina JE, Califano JA, Wolf GT, Ferlito A, Som PM, Day TA (2008) Consensus statement on the classification and terminology of neck dissection. Arch Otolaryngol Neck Surg 134(5):536–538 10.1001/archotol.134.5.536 18490577

[16] Adam MA, Pura J, Gu L, Dinan MA, Tyler DS, Reed SD, Scheri R, Roman SA, Sosa JA (2014) Extent of surgery for papillary thyroid cancer is not associated with survival: an analysis of 61,775 patients. Ann Surg 260(4):601–607 25203876 10.1097/SLA.0000000000000925 PMC4532384

[17] Hughes DT, Rosen JE, Evans DB, Grubbs E, Wang TS, Solórzano CC (2018) Prophylactic Central Compartment Neck Dissection in Papillary Thyroid Cancer and Effect on Locoregional Recurrence. Ann Surg Oncol 25(9):2526–2534 29786126 10.1245/s10434-018-6528-0

[18] Chen L, Wu YH, Lee CH, Chen HA, Loh EW, Tam KW (2018) Prophylactic Central Neck Dissection for Papillary Thyroid Carcinoma with Clinically Uninvolved Central Neck Lymph Nodes: A Systematic Review and Meta-analysis. World j surg 42(9):2846–2857 29488066 10.1007/s00268-018-4547-4

[19] Machens A, Hauptmann S, Dralle H (2009) Lymph node dissection in the lateral neck for completion in central node-positive papillary thyroid cancer. Surgery 145(2):176–181 19167972 10.1016/j.surg.2008.09.003

[20] Miyauchi A, Kudo T, Ito Y, Oda H, Sasai H, Higashiyama T, Fukushima M, Masuoka H, Kihara M, Miya A (2018) Estimation of the lifetime probability of disease progression of papillary microcarcinoma of the thyroid during active surveillance. Surgery 163(1):48–52 29103582 10.1016/j.surg.2017.03.028

[21] Tong M, Li S, Li Y, Li Y, Feng Y, Che Y (2019) Efficacy and safety of radiofrequency, microwave and laser ablation for treating papillary thyroid microcarcinoma: a systematic review and meta-analysis. Int J Hyperthermia 36(1):1278–1286 31826684 10.1080/02656736.2019.1700559

[22] Asari R, Koperek O, Scheuba C, Riss P, Kaserer K, Hoffmann M, Niederle B (2009) Follicular thyroid carcinoma in an iodine-replete endemic goiter region: a prospectively collected, retrospectively analyzed clinical trial. Ann Surg 249(6):1023–1031 19474675 10.1097/SLA.0b013e3181a77b7b

[23] Zhao X, Wang JR, Dadu R, Busaidy NL, Xu L, Learned KO, Chasen NN, Vu T, Maniakas A, Eguia AA, Diersing J, Gross ND, Goepfert R, Lai SY, Hofmann MC, Ferrarotto R, Lu C, Gunn GB, Spiotto MT, Subbiah V, … Zafereo ME (2023) Surgery After BRAF-Directed Therapy Is Associated with Improved Survival in BRAF^V600E^ Mutant Anaplastic Thyroid Cancer: A Single-Center Retrospective Cohort Study. Thyroid 33(4):484–491 36762947 10.1089/thy.2022.0504 PMC10122263

